# Nanofiber-Based Face Masks and Respirators as COVID-19 Protection: A Review

**DOI:** 10.3390/membranes11040250

**Published:** 2021-03-30

**Authors:** Wafa K. Essa, Suhad A. Yasin, Ibtisam A. Saeed, Gomaa A. M. Ali

**Affiliations:** 1 College of Science, University of Duhok, Duhok 42001, Iraq; wafa.k.essa@uod.ac (W.K.E.); ibtisamsaid@uod.ac (I.A.S.); 2 Chemistry Department, Faculty of Science, Al-Azhar University, Assiut 71524, Egypt

**Keywords:** air pollutants, COVID-19, face masks, respirators, nanofibers filter media

## Abstract

Wearing face masks, use of respirators, social distancing, and practicing personal hygiene are all measures to prevent the spread of the coronavirus disease (COVID-19). This pandemic has revealed the deficiency of face masks and respirators across the world. Therefore, significant efforts are needed to develop air filtration and purification technologies, as well as innovative, alternative antibacterial and antiviral treatment methods. It has become urgent—in order for humankind to have a sustainable future—to provide a feasible solution to air pollution, particularly to capture fine inhalable particulate matter in the air. In this review, we present, concisely, the air pollutants and adverse health effects correlated with long- and short-term exposure to humans; we provide information about certified face masks and respirators, their compositions, filtration mechanisms, and the variations between surgical masks and N95 respirators, in order to alleviate confusion and misinformation. Then, we summarize the electrospun nanofiber-based filters and their unique properties to improve the filtration efficiency of face masks and respirators.

## 1. Introduction

World population growth, industrialization, and urbanization have initiated the production of enormous quantities of contaminants being emitted into the air, with no notion of how they could affect human health. Recently, air contaminant concentrations have risen above Air Quality Guidelines (AQG) issued by the World Health Organization (WHO) in many developed countries, leading to environmental protection policies for all individuals across the world. Air pollution severely destroys life quality and poses an immediate danger to public health [1]. Symptoms, such as weeping, coughing, angina, and difficulty breathing, are related to air pollution immediately after exposure, and may also cause more subtle, long-term harm to human health. People are typically ignorant about the impacts of long-term exposure to their health (as well as the fact that long-term exposure may worsen their medical conditions). Air pollution accesses the human body via the respiratory tract, and it also has systemic influences that can harm several organs [2,3].

In metropolises, because of these severe environmental problems, people wear masks or respirators for filtering polluted outdoor air, and air filtration equipment is becoming more common indoors as well. Indeed, using reception-based solutions via improving masks and respirators as effective means to capture hazardous particulates [4]. Air filtration is a promising, efficient, and practical technique used against air pollutants. Even now, extensive efforts are employed to enhance highly efficient air filter media, with a focus on improving filter efficiency [5,6,7,8]. Although conventional air filtration media, such as high-efficiency particulate air (HEPA) filters, have a high efficiency of filtration (of approximately 99.97%) for airborne particles (0.1–0.5 μm), their performance is still low for particulate matter (PM) in the sub-micrometer. Another drawback of using thicker filtering media is the high-pressure drop or energy costs to offset the resulting flow resistance. Such disadvantages of traditional high-performance filters can be mitigated using an electrospinning technique to prepare nanofiber-based filters. Carbon-based air filters are designed to trap air pollutants and fabricated as protective masks. Because of their high surface area, abundance, stable chemical structure, low resistance, and high functionalization ability with other materials, carbon materials are promising candidates for air purification. Particularly, since the diameter of carbon nanofibers is comparable to the free path of the air molecules (66 nm under normal conditions), they overcome the inherent problem between filtration efficiency and pressure drop [9,10]. They can be used to remove volatile organic compounds, nanoparticles, and bacterial contaminants in the air [10].

Electrospinning permits fiber production, with nanoscale diameters varying between 40 and 2000 nanometers as excellent candidates for biomedical applications [11,12]. In addition, electrospun nanofiber filters possess a high ratio of surface area/volume that significantly increases the possibility of pollutant deposition on the surface of the fiber, and consequently develops the performance of the filter with a relatively low-pressure drop [13]. This review is intended to condense previous research into a concise, easy-to-read document, focusing on the efficacy of face masks and respirators containing nanomaterials in their structures.

## 2. Air Pollutants and Adverse Health Effects

Air pollution is one of the most earnest threats to the environment, and it also has an adverse impact on human health. The World Health Organization (WHO) announced that diseases resulting from household air pollution (indoor) cause the death of approximately 4 million people each year; moreover, 7.6% of all deaths were caused by ambient air pollution (outdoor) universally in 2016 [14]. Household (indoor) air pollutants, such as environmental tobacco smoke (ETS), along with what is generated by the combustion of biomass fuel (dung, wood, etc.), are also disturbing in various regions [15]. Air pollutants of ambient (outdoor) areas are a combination of thousands of components. From a health viewpoint, PM and pollutants such as ozone (O_3_), volatile organic compounds (VOCs), nitrogen dioxide (NO_2_), carbon monoxide (CO), sulfadiazine, and sulfur dioxide (SO_2_) are the most important among them [16,17,18].

**Figure 1:**
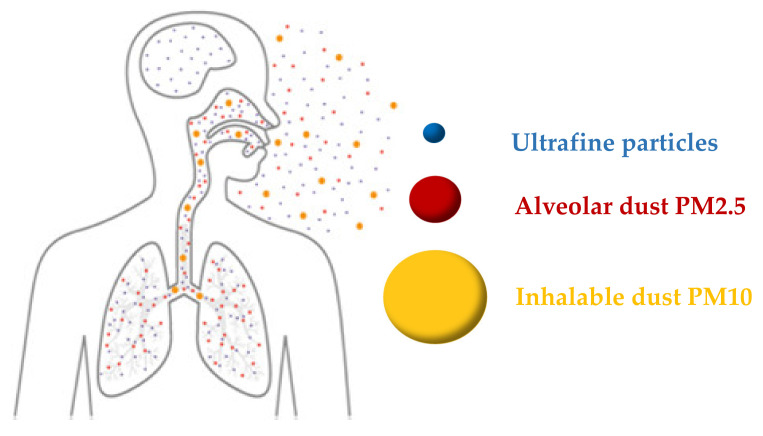
Different sizes of particulate matter (adapted with permission from Reference [26]).

Primary pollutants are released immediately into the air by fossil fuel combustion (similar to nitrogen and sulfur oxides, and soot particles). Industrial sources, motorized road traffic, residential heating, and power generation are the primary PM sources. Once primary pollutants interact in the atmosphere, secondary pollutants are produced, including O_3_, PM, and aerosol [19]. PM is the sum of particles suspended in the air, such as liquid droplets and solid particles. PM is classified according to the particle size as large (PM_10_), fine (PM_2.5_), and ultrafine (PM_0.1_), where the subscript represents the upper limit of particulate diameter in micrometers (Figure 1). Ultra-fine particles are invisible in contrast to particles being large, visible as haze, or dust with sufficient lighting.

Upper airways and mucous membranes might be affected by large PM_10_ particles, resulting in coughing and tears. PM_2.5_ and PM_0.1_ cause the worst health influences because they can get to the pulmonary alveoli and pass into the circulatory system, causing severe health problems and increase morbidity and mortality in long-term exposure [20]. Several reports have correlated ultrafine particle exposures to various symptoms, particularly respiratory and cardiovascular diseases [21,22,23,24]. Additionally, some natural pollutants, including bacteria, pollen, and certain microorganisms and aerosols carrying viruses, cause respiratory infectious diseases, for instance, chronic obstructive pulmonary disease (COPD), cancer of the lung, or asthma [25].

**Figure 2:**
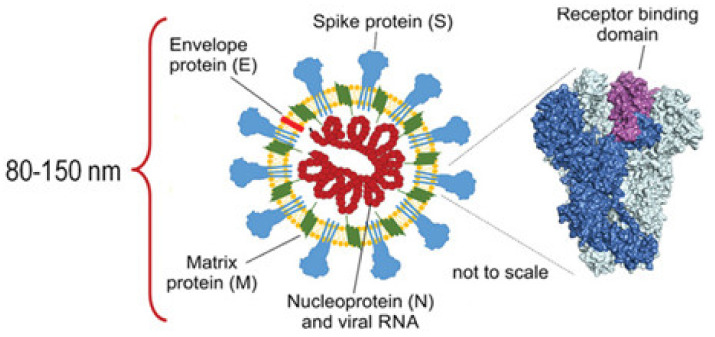
Coronavirus disease (COVID-19) (adapted with permission from Reference [35]).

The world is currently—during the ongoing pandemic of coronavirus disease—facing dangerous viral aerosols, brought about by severe acute respiratory syndrome coronavirus 2 (SARS-Co-2) [27,28]. The microsize aerosols (0.25–1.0 μm in diameter) that carry the virus are released into the air once the infected person sneezes, coughs, and breaths [29,30]. The released droplets significantly vary in number and size; during a sneeze, up to 40,000 droplets are released at a speed of 100 m/s [31], and approximately 3000 droplet nuclei are generated during a cough [32]. The size of the COVID-19 virus (Figure 2) is about 80–150 nm; the small size of SARS-CoV-2 led to concern because it could allow the virus to pass through respirator filters tested for larger particles of 0.3 μm [33]. Because this virus has a long incubation period (3–20 days), and there are asymptomatic carriers, wearing a face mask or respirator, social distancing, and paying attention to personal hygiene are encouraged to prevent spreading the virus [34].

## 3. Face Masks and Respirators

Filtering facepieces (FFP), face masks, and respirators are cost-effective, beneficial, and practical due to their good performance at reducing exposure to airborne particulate matter [34]. Face masks are disposable, loose-fitting devices that provide physical barriers to separate the wearer’s mouth and nose from potential pollutants in the surrounding environment. A regular face mask comprises one or two layers of plastered or flat fabric, typically made of paper or cotton. It is typically only efficient in catching large particles of pollutants and is not used for preventing infectious diseases.

The most commonly used surgical mask comprises of polypropylene (PP) of 3-ply layers (at least), with different thicknesses and capabilities for protecting the wearer from infectious particles. It should have 80% bacteria filtration efficiency, at minimum; however, we should note that it does not provide reliable protection against small airborne particles and viruses [36,37]. Surgical masks should not be shared with others and are labeled as masks for surgery, dental, isolation, or medical procedures. They can come with or without a face shield. If worn correctly, surgical masks are designed for protection against air pollution in a sterilized field, or in a working environment—for protection against large particles, such as spit and mucous generated from the wearer. Another usage is to minimize the risk of splashed or sprayed body fluids, blood, and secretions from reaching the wearer’s nose and mouth [38]. Since face masks do not have sufficient filtering to protect the wearer from respiratory droplets and do not prevent leakage around the mouth after inhalation due to loose-fitting, they are used for one time only [39].

On the other hand, respirators are particular types of personal protective equipment (PPE) designed to protect the wearer from inhaling harmful airborne particles (including infectious agents, such as coronavirus, SARS, H1N1, etc.), gases, or vapors [40,41]. They are usually pre-molded, fit tightly, adhere with an elastic band to the head, and utilize filters to reduce inhaled harmful air contaminants. Respirators are categorized into air-purifying and supplied respirators with filtering devices and breathing apparatuses, respectively. In particular, respirators help reduce the wearer’s airway exposure to inhalable pollutants with a size of fewer than 100 μm. A valved respirator enables it to exhale air easier, is more convenient for wearing, and contains less moisture build-up within the respirator. The problem of ventilators with valves is that they filter the air in (inhale) but not the air out (exhale). Regarding COVID-19, the one-way protection of valved respirators places individuals around the wearer at risk; for such a reason, hospitals do not use respirators with valves [42].

## 4. Standards for Face Masks and Respirators

Face masks and respirators are subject to specific standards and regulations, based on the nation or geographical region (Table 1). The Food and Drug Administration (FDA) cleared the surgical masks in the United States, and they should be complying with the standards of the American Society for Testing and Materials (ASTM). The ASTM F2100-11 standards are certified with five performance metrics for materials used to make medical face masks: resistance to fluid, breathability, bacterial filtration efficiency (BFE), particulate filtration efficiency (PFE), and flammability. Depending on their test marks, ASTM attributes the substance’s barrier efficiency to a numerical rating: level 1 barrier—fluid exposure at low risk; level 2 barrier—fluid exposure at moderate risk; level 3 barrier—fluid exposure at high risk. Surgical masks in Europe should be standard by European Norm (EN). According to the EN 14683 standard for surgical masks, the three types of surgical mask effectiveness are: type I or BFE1—more than 95% bacterial filtration efficiency, type II or BFE2—more than 98% bacterial filtration efficiency, type IIR—bacteria filtering effectiveness of more than 98% and splash-resistant. The European standard added a resistance test for types IR and IIR; IIR has the most resistance.

Respirators are tested and cleared via the National Institute for Occupational Safety and Health (NIOSH) in the U.S., which belongs to the Centers for Disease Control and Prevention (CDC). The respirator series, air-purifying types, N (not oil resistant), R (resistant to oil), and P (oil proof) are approved by NIOSH under 42 Code of Federal Regulations (CFR) Part 84, each at 95, 99, and 99.97% filtration efficiency levels, as shown in (Table 1) [43]. Among them, the N95 respirator is the most extensively used [44]. By EN 149:2001, the Legislation of European Standards for respirators is covered. There are three types of disposable respirators according to that standard: 80% as low efficiency, or FFP1; 94% as medium efficiency, or FFP2; and 99% as high efficiency, or FFP3, as shown in Table 1 [45]. The higher the FFP number, the more protection the respirator can offer if adequately used. EN 149:2001 includes breathing resistance, filter penetration, flammability, extended exposure (loading), dolomite dust clogging (optional), and total inward leakage (TIL). Since the standard N95 and FFP3 or FFP2 respirators are approximately equivalent, they are recommended to be used for prevention of airborne infectious diseases [46].

## 5. The Variations between a Surgical Mask and N95 Respirator

**Figure 3:**
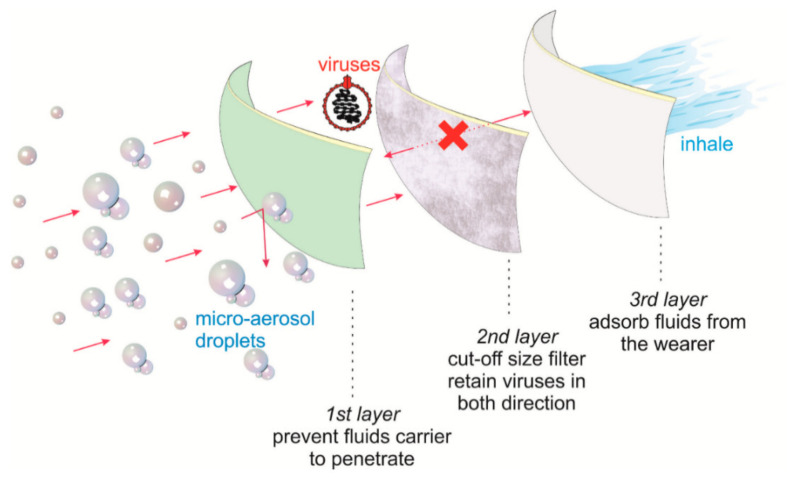
Structure of the surgical face mask (adapted with permission from Reference [48]).

**Figure 4:**
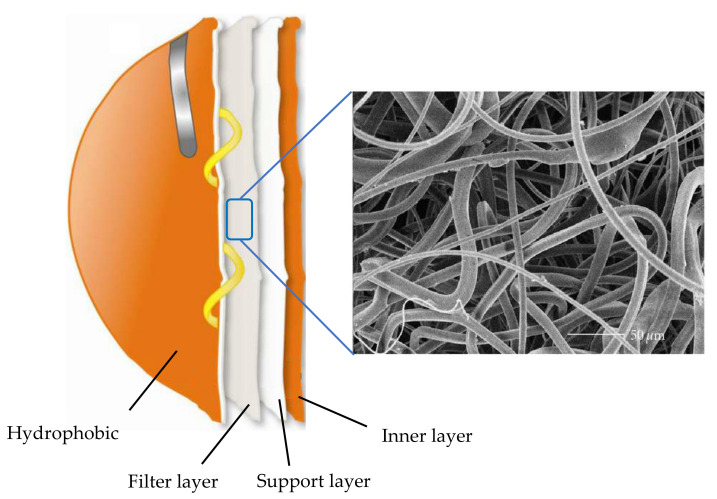
Structure of N95 respirator (adapted with permission from Reference [49]).

With the rapid emergence of infectious diseases, such as COVID-19, there has been significant interest in using surgical masks and N95 respirators as part of infection prevention procedures. The surgical masks consist of very fine middle layers with extra fine glass fibers, which are covered on both sides by acrylic bonded parallel-laid or wet-laid nonwoven material (Figure 3). N95 respirators are engineered for specific functions—different from surgical masks (even though they often appear to be identical) (Figure 4) [47]. The N95 consists of an outer layer constructed of hydrophobic nonwoven PP (to prevent moisture), a filter layer of melt-blown nonwoven PP (to capture oil and non-oil-based particles), a support layer, and an inner layer, as shown in Figure 4.

## 6. Filtration Mechanisms of Particles

**Figure 5:**
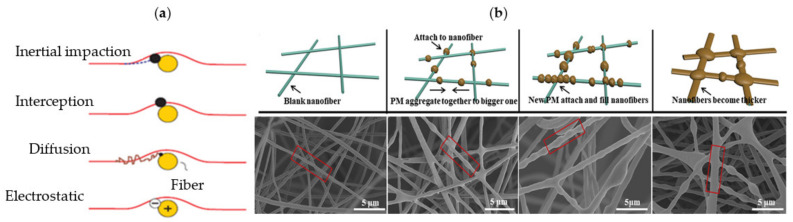
Mechanisms of particle filtration: (**a**) five collections of mechanism, and (**b**) interaction of PM with nanofibers (adapted with permission from Reference [50]).

Face masks and respirators have commonly been used as protective devices for filtering airborne contaminants. Fibrous filters are used in current surgical masks and respirators, made from several flat, fine, fiber layers (μm in diameter) of nonwoven mats, capable of capturing PM particles through physical adhesion barriers. Various parameters regulate filtration effectiveness, such as fiber diameter, porosity, and filter thickness. The filtration mechanism is a significant aspect in terms of the accuracy and efficiency of the filter media. Filtering of particles is essentially performed through five collections of mechanisms: (1) interception, (2) inertial impaction, (3) diffusion, (4) gravitational settling, and (5) electrostatic attraction (Figure 5a). On the other hand, deep filtration with low efficiency and a longer life occurs when microfiber is used, while the nanofibers lead to high efficiency, a shorter life, and a surface filtration process. To perform deep, high efficiency and a shorter time filtration process, the beaded nanofiber is recommended to be used (Figure 5b).

Generally, all collection mechanisms, except the electrostatic attraction, refer to mechanical filters and are affected by particle size and velocity. Interception and inertial impaction are commonly known to be predominant combination mechanisms for macro and microparticles (>0.3 μm), while diffusion is predominant for nanoparticles (<0.3 μm). An interception occurs when the particles follow streamline round the fiber and come into contact with the fiber’s surface, and deposit on it because of van der Waals forces. Inertial impaction occurs when the particle changes its streamline direction near a filter fiber and impacts the fiber due to inertia. This mechanism is more efficient for capturing large particles and increases at higher particle velocity. On the contrary, particles under 0.3 μm are mainly affected by diffusion. These very tiny particles move across streamlines (Brownian motion) until they contact the fiber, because of air molecules’ random movements. In gravitational settling, and due to gravity, large particles may settle in slow movement airstreams. The electrostatic attraction may be significant, but hard to measure because it needs to know the fiber charges and particles. Through the Columbia attraction, particles that are charged are attracted to the fibers oppositely charged [50,51]. When the filter fibers are in the nanoscale, the filtration conditions can change. Airflow aerodynamic behavior around the periphery of nanostructured fibers will significantly change. In addition, the strong forces of van der Waals that are capable of adsorbing submicron-sized particles will be produced. Due to the pores’ good interconnectivity, the diffusion, inertial effect, and interception will also be enhanced [52].

## 7. The Composition of Surgical Masks and N95 Respirators

The filtering materials of face masks and respirators are made of nonwoven fabric, considered disposal after use because their reuse significantly degrades their filtering performance. The salient benefit of nonwoven technology concerns the potential to manufacture fabrics and structures that cost much lower than other fabric technology, such as woven and knitted. Most surgical mask industries use spunbond melt-blown spunbond (SMS) technology for producing surgical masks. The suitable polymer materials for surgical mask manufacturing are PP, polyethylene terephthalate (PET), polystyrene, polycarbonate, polyethylene (PE), polyester, etc. [53,54,55,56]. PP is usually used to produce surgical masks by fabricating spunbond nonwoven layers (20 g/m^2^) and melt-blown nonwoven sheets (25 g/m^2^) [57]. It is relatively cheap and has low melt viscosity for easy processing. In addition, these polymers are transparent, lightweight, and provide high-optical clarity; thus, they could be three-dimensionally (3D) printed as face masks for COVID-19 protection [58].

A standard surgical mask is usually comprised of three layers: a soft nonwoven absorbent (layer being inner), a melt-blown (the layer at middle), and a nonwoven hydrophobic (layer being outer). Each layer has a specific function: the inner layer is purposed to absorb moisture, sweat, and the spit of the wearer; the middle layer of the surgical mask is designed as an electret filter to prevent germs from coming in or exiting from the mask; and the outer layer is purposed to repulse water, bodily fluids, and blood. Masks are manufactured by machines where the layers are ultrasonically welded together, and the masks are labeled with ear strings and nose strips. Masks are first sterilized before being exported.

The N95 respirator is comprised of many layers of PP nonwoven fabric. The two external protective layers are produced using a spunbond to cover both the inner and outer of the N95 respirator. There is a layer of pre-filtration between these spunbond layers, which may be as thick as 250 g/m^2^, making it stiffer and thicker, so it can be flexible enough to form the required shape. The last layer is a nonwoven melt-blown electret material of high-quality that controls filtration competence. The full respirators are manufactured by converted equipment, welding the layers by ultrasonic and adding belts and strips of metal to regulate the mask over the user’s face. Finally, respirators are sanitized before shipment.

## 8. Electrospun Nanofibers and Their Applications in Face Masks and Respirators

**Figure 6:**
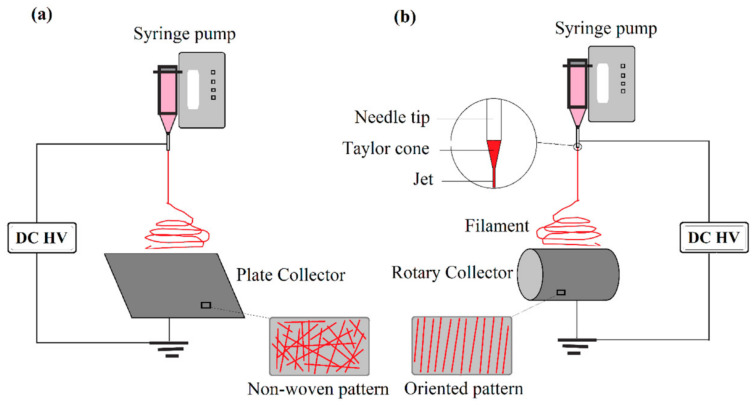
Electrospinning process (adapted with permission from Reference [65]).

Electrospinning is a novel technique to manufacture nanofibers, as it provides a quick procedure, low expense, and precise control of the nanofiber compositions and geometric features. In electrospinning, high voltages apply to melts or polymer solution droplets to eliminate the tension of liquid surface and ultrafine fibers with diameters between 40 and 2000 nm to be created (Figure 6) [59]. Selecting a suitable solution concentration, appropriate voltage, and the space between the supporting collector and the syringe tip is of considerable importance for synthesizing uniform nanofibers. As an essential part of this technology, nanofiber-based filter media are the main components for enhancing filtration performance [60,61,62,63,64].

Electrospun nanofiber-based filter media possess a high ratio of surface/volume, low-pressure drop, good interconnectivity of voids, and controllable connectivity and morphology, rendering them desirable to achieve excellent filtering. Because of its fragility, electrospun nanofibers do notcan not be used individually at filter media, it should be deposited onto a substrate, usually fabric as nonwoven. Glass, polyester, nylon, and cellulose are the common substances used to support the electrospun nanofibers. The substrate should have excellent mechanical properties to enable pleating, fabrication of filter, and toughness in usage [66]. For the filtration propose, substrates are selected for pleating, filter fabrication, durability in use, and filter cleaning.

Currently, most researchers who are interested in the air filter industry are searching for technology based on nanofibers to enhance dust interception capability and filtration quality. There are already several applications of commercialized filters, as well as those in progress. Using nanofibers in face masks and respirators is better than the available commercialized. The active filters used in commercial face masks and respirators right now employ small diameter PP fibers in the range of 500–1000 nm; these filters achieve filtration with the help of static electricity. The pore size decreases as the fiber diameter decreases, and the distribution of fibers per unit area becomes denser. The electrostatic assisted melt-blown improves filtration quality by creating a small charge in the fabric, which increases the fabric’s adsorption capability.

However, such filters can lose their static electricity after wearing for an extended period and when exposed to water, thereby reducing their filtration efficiency, so this type of filter is designed to be disposable. This is not the case for nanofibers that do not depend on static electricity to filter contaminants; they use smaller pores and reasonable distribution of pores to physically filter aerosols that contain harmful dust or viruses [67].

Several studies and patents on nanofibers have been identified in different face masks and respirator applications [68,69,70]. Munzarová (2013) developed barrier fabrics based on nanofibers via electrospinning to be laminated onto face masks. This barrier protects from the permeation of microorganisms, dust particles, and allergens [71]. Skaria and Smaldone (2014) produced a prototype nanofiber-based filter media fitted face mask compared to the N95 respirator. They found that the prototype significantly reduced airflow resistance, resulting in greater face mask compliance and increased filtration efficiency, similar to that obtained when using an N95 respirator [72].

With a slightly different perspective, Li and Gong (2015) informed of the development of nanofiber-based on polysulfone for mask filtration, utilizing electrospinning to be coated onto nonwoven PP, aiming to avoid the inhalation of harmful pollutants in contaminated haze air. The nanofiber mat thickness was modified at different collective preparation periods (15 min < 30 min < 60 min), and these three nanofiber masks were compared with nonwoven disposable face masks, nonwoven operative room masks, N95 and R95 respirators, and Ito PM_2.5_. It was observed that electrospun nanofiber masks might be efficient at filtering out PM_2.5_ particles and, at the same time, maintain good breathability [73]. Similarly, Akduman (2019) prepared a nanofiber layer of cellulose acetate (CA) and polyvinylidene fluoride (PVDF) with 100% mechanical filtration for face masks and respirators capable of meeting the specifications of N95 respirators. The effect of nanofiber mat thickness, nanofiber diameters, and pore size on filtration efficiency was compared [38,74]. The mean diameter of PVDF nanofibers (236.50 nm) was smaller than the diameter CA (319.02 nm) nanofibers. Therefore, CA nanofibers showed better filtration efficiency [74].

The use of solution blow spinning (SBS) nanofibers is a significant step in developing a composite mask [75,76]. Noel et al. (2019) used the SBS nanofibers method in composite multilayered filter masks; they prepared three different nanofiber fabrics types, cellulose diacetate (CDA), polyacrylonitrile (PAN), and PVDF. They demonstrated that the presence of functionalities of different molecules in the electrospun nanofibers had a significant effect on the efficiency of filtration, i.e., PAN nanofiber had the best filtration efficiency (0.05 Pa^−1^) of the quality factor and good air permeability, whereas, among all the nanofibers studied, PVDF air filter quality was the lowest, with (0.02 Pa^−1^) of the quality factor [77].

Moreover, titanium dioxide (TiO_2_), carbon nanotubes (CNTs), and silver (Ag) have been easily used as additional materials for coating onto electrospun nanofibers. Nanostructured TiO_2_ was of considerable interest to different coating materials because of its remarkable catalysis of UV rays and shielding properties [78,79]. Ruan et al. (2020) fabricated and developed the polyacrylonitrile-co-polyacrylate (PAN-co-PMA):TiO_2_ membrane of the electrospun nanofiber [80]. The electrospun nanofiber membrane features, such as permeability of air, PM trapping, and aerosol inspection, were evaluated methodically. For two types of nanofiber membranes, the microfiber nonwoven, the nanofiber membrane, and the nonwoven fabric bracket were built-up into a multi-layer structure electrostatic force. The PAN-co-PMA:TiO_2_ nanofiber membrane bonding system demonstrated effective PM_2.5_ removal and superior air permeability (284–339 mm/s) [80]. Several studies have manifested the use of activated carbon, and carbon nanofiber (AC/CNF) composite was found to be a suitable alternative for the respirator cartridge due to being lightweight and its appropriate absorption ability [81]. In the study by Jahangiri et al. (2013), the granulated (AC/CNF) was utilized to absorb and remove VOCs from breathing air in the respiratory mask cartridges. The findings demonstrated that the breakthrough period was longer for this cartridge than for other types [82].

It is known that incorporating antimicrobial agents, such as silver, with nanofibers, exhibits antimicrobial properties in the filters [83]. They were mainly distributed on the nanofiber surface. Microorganisms can be killed when they contact silver nanoparticles during filtration [84], for example, nanosilver-embedded polyacrylonitrile nanofibers [85]. Yang et al. (2017) demonstrated the thermal management effect in the nano-fiber-based face mask with a nylon 6/nano PE model system that manifests high efficacy for PM_2.5_ capturing (99.6%) with lower pressure drop [62]. Moreover, they modified the nano PE substrate with silver. The fiber/Ag/nano PE mask filter reveals a value of (87.0%) as IR reflectance is high and might be utilized in winter or summer to protect the wearer from contaminated air and render the face warm or cool/comfortable [62].

Additionally, for protection against bacteria and viruses, nanofibers comprising superabsorbent polymers (SAPs) have been produced in order to provide greater convenience, adding additional functions, as well as medical care. To this end, many researchers have fabricated electrospun superabsorbent nanofibers to increase material absorption ability, to be utilized as personal hygiene products, microbe bio-filters, and disposable face masks [86,87]. Sivri (2018) used nanofibers electrospun of (PVA/SAP) aqueous polymer solutions to be coated onto face masks for developing virus barrier functions and liquid absorption functions. It was found that all face masks were successfully coated with nanofiber, according to Fourier transform infrared spectroscopy (FTIR) investigations and scanning electron microscopy (SEM). Air permeability and capacity to absorb liquids showed that the coating with nanofiber improved the face mask’s hydrophilicity while permeability of air decreased reversely [88].

The coronavirus pandemic outbreak has prompted a lack of face masks and respirators in the world. Therefore, there is an immediate need for a secure disinfection method, and reuse them, with minimum loss of efficiency and integrity [89,90,91,92]. Lee et al. (2019) developed high-performance membrane filters of polybenzimidazole (PBI) nanofiber that can be utilized for dustproof masks or other air filters. They indicated that the PBI nanofiber filter membrane achieved high filtration efficiency (~98.5%) at a significantly lower pressure drop (130 Pa), in contrast to the commercial face mask. They also demonstrated the PBI filter reusability membrane, due to its thermal, mechanical, and chemical stability, after the proposed cleaning process [93]. An injection molded autoclavable, scalable, conformable (iMASC) system was designed and produced by James et al. (2020) for aerosol-based protection N95 content filters that can be installed and replaced as desired. To understand the masking potential with various face forms and sizes, the finite element (FE) analysis tested the deformability of the iMASC system. The iMASC system has been shown to match several various face shapes and sizes with success, utilizing a test method confirmed by Occupational Safety and Health Administration (OSHA). These data support more qualification tests required for use in the healthcare sector [94].

Nazek et al. (2020) have improved a nanoporous flexible Si-based template on a silicon-on-insulator (SOI) wafer utilizing potassium hydroxides (KOH) etching, utilizing the template as a hard mask through a reactive ion etching process for transferring patterns onto a lightweight (<0.12 g) and flexible polymeric membrane. The flexible membrane might be utilized on the N95 mask as reusable to boost its filtration efficiency against particles sub-300 nm, including COVID-19. Furthermore, N95 mask reusability contributes toward eliminating the challenges surrounding single-use face mask shortages [95].

## 9. Conclusions

The COVID-19 outbreak has become a serious problem in modern human history. Wearing protective face masks, preserving personal hygiene, and social distancing should be followed to help prevent the spread of COVID-19. This disease has led to a worldwide increase in the usage of billions of face masks and respirators every day, resulting in a high demand for goods that produce them. In general, most commercial filtering facepieces use electrostatic filter media that can degrade over time (due to many different variables). Nanotechnologies play a crucial role in this issue by fabricating nanomaterials with special characteristics for air filtration. A nanofiber-based mask would not lose its efficiency in time or due to many different factors (because of its mechanical filtration efficiency protection, from the mask layers). In this regard, we summarize the filters based on electrospun nanofibers and their unique characteristics to increase filtration performance of face masks and respirators.

## Figures and Tables

**Table 1:** American Society for Testing and Materials and European Norm standards for face masks and respirators.

FFP Type	Standards	Filtration Efficiencies		
Surgical Mask	USA: ASTM F2100-11	Level 1	Level 2	Level 3
	standard	95%	98%	98%
	EN:	Type I	Type II	Type IIR
	EN 14683 standard	95%	98%	98%
Respirator	USA: NOISH 42 CFR Part	N95/R95/P95	N99/R99/P99	N100/R100/P100
	84	95%	99%	99.97%
	EN:	FFP1	FFP2	FFP3
	EN 149:2001	80%	94%	99%

ASTM = American Society for Testing Materials. FFP = Filtering Facepieces. NIOSH = Respirators are tested and cleared via the National Institute for Occupational Safety and Health. CFR = Code of Federal Regulations.

## Data Availability

The data presented in this study are cited (reference numbers).
